# SES DISPARITIES IN FUNCTIONAL IMPAIRMENT WITHIN OLDER ASIAN AMERICANS: COMPARISON OF FOUR RACIAL/ETHNIC GROUPS

**DOI:** 10.1093/geroni/igad104.0482

**Published:** 2023-12-21

**Authors:** Katherine Wang, Zheng Zhu, Xiang Qi

**Affiliations:** Duke University, Durham, North Carolina, United States; New York University, New York City, New York, United States; New York University, New York City, New York, United States

## Abstract

This study investigated to what extent socioeconomic status (SES) disparities associates with functional impairment within older Asians in comparison with other racial/ethnic groups. Data were from the National Health and Nutrition Examination Survey 2011-2018 that included 3,297 White, 1,755 Black, 1,708 Hispanic, and 730 Asians aged ≥60. Physical functioning was measured by activities of daily living (ADL) or instrumental activities of daily living (IADL). Memory was determined by CERAD-WL, and language fluency was measured by Animal Fluency Tests. We conducted multivariate logistic regressions to examine the association between SES and functioning impairment within racial/ethnic groups and performed seemingly unrelated regressions to compare the regression coefficients across subgroups. For the age- and sex-adjusted prevalence of functional impairment, Asians with ≤high school education had the highest prevalence of memory impairment among all races/ethnicities, and no difference was observed for all racial/ethnic groups with >high school education. After adjustment for key covariates, Blacks and Hispanics had higher odds of ADL/IADL disabilities and cognitive impairment relative to Whites. The odds of ADL/IADL disabilities did not differ with Asians and Whites, whereas Asians were more likely to have language fluency impairment than Whites. Education disparity for ADL disability (OR, 3.42; 95% CI, 2.21-5.29) and memory impairment (OR, 16.87; 95% CI, 8.57-33.21) were largest among Asians compared to Whites, Blacks, and Hispanics. Income disparity for function impairment did not significantly differ across four racial/ethnic groups (all P > 0.05). This study indicates that Asians fare worst in the burden of functional impairment due to education disparity.

